# Paravertebral nerve block versus combined serratus anterior plane and intercostal nerve blocks in thoracoscopic lung segment surgery in elderly: a retrospective study

**DOI:** 10.1186/s13019-025-03662-w

**Published:** 2025-10-24

**Authors:** Meng Xia, Runfeng Wang

**Affiliations:** 1 Department of Anesthesiology, Affiliated Nanjing Brain Hospital, Nanjing Medical University, 264 Guangzhou Road, Nanjing, 210029 Jiangsu China; 2 ICU,Affiliated Nanjing Brain Hospital, Nanjing Medical University, 264 Guangzhou Road, Nanjing, 210029 Jiangsu China

**Keywords:** Thoracoscopic surgery, Serratus plane block combined with intercostal nerve block, Thoracic paravertebral

## Abstract

**Objective:**

In order to accelerate the rapid recovery of elderly patients undergoing lung surgery, we tend to use multimodal analgesic protocols. A retrospective analytical study was used to analyse postoperative analgesic regimens suitable for older adults.To compare the efficacy of ultrasound-guided paravertebral nerve block and anterior serratus planar nerve block combined with intercostal nerve block for postoperative analgesia in elderly patients undergoing thoracoscopic segmental lung resection.

**Design:**

Elderly patients undergoing elective thoracoscopic segmental lung resection in our hospital were reviewed and divided into the paravertebral nerve block group (Group T) and the anterior serratus plane nerve block combined with intercostal nerve block group (Group S).

**Setting:**

Department of Anaesthesiology, Brain Hospital (Chest Campus), Nanjing Medical University, China.

**Interventions:**

In both groups, the appropriate nerve block was performed under ultrasound guidance while the patients were lying on their sides after induction of anaesthesia.

**Measurements & main results:**

The primary outcomes of interest were the Visual Analogue Scale (VAS) scores for pain at rest and motion, assessed at preoperative (T0), 24 h postoperative (T1), 48 h postoperative (T2), and 72 h postoperative (T3) for both groups. Additionally, the number of postoperative self-controlled compressions and the number of rescue analgesia administrations within the first 48 h postoperatively were recorded.The primary endpoints of the study are as follows: The resting and motion VAS scores of the two groups were compared at each time point, and the resting and motion VAS scores of the two groups at T1, T2 and T3 were higher than those at T0, and the differences were statistically significant (*P* < 0.05),and there was a difference in the number of postoperative remedial analgesia in group T compared with group S (*P* < 0.05).

**Conclusion:**

Both ultrasound-guided thoracic paravertebral nerve block and anterior serratus plane block combined with intercostal nerve block can provide effective postoperative analgesia for elderly patients undergoing thoracoscopic pulmonary segmentectomy, serving as safe and feasible multimodal analgesia protocols.Compared to paravertebral nerve block, the anterior serratus plane block combined with intercostal nerve block is advantageous due to its relative ease of application.

## Introduction

Along with the aging of the population and the improvement of people’s health awareness, the proportion of elderly patients undergoing chest CT health checkups has increased, and the number of elderly patients requiring lung surgery is increasing. Thoracic surgery, due to its complexity, trauma, and significant postoperative pain, poor postoperative pain control can seriously affect the recovery of patients, especially for elderly patients. With the emergence of minimally invasive thoracic surgery, especially single-port thoracoscopic technology [1], perioperative analgesia for thoracic surgery has evolved to a less opioid mode, and thoracic nerve blocks are increasingly widely used [2, 3]. Paravertebral nerve block, erector spinae plane nerve block, anterior serratus plane nerve block and intercostal nerve block have definite perioperative analgesic effects in thoracic surgery, and can reduce the amount of perioperative opioids to a certain extent, among which, paravertebral nerve block has comparable analgesic effects with thoracic segmental epidural nerve block, and the operation is more simple, with high safety, and it has gradually become the gold standard for perioperative analgesia in thoracic surgery [4, 5]. Whether anterior serratus plane nerve block combined with intercostal nerve block has the same analgesic effect as paravertebral nerve block in thoracoscopic lung surgery is still controversial [6]. We retrospectively analysed the clinical outcomes of ultrasound-guided anterior serratus plane nerve block combined with intercostal nerve block versus paravertebral nerve block in elderly patients undergoing thoracoscopic segmental lung resection at our institution.

## Information and methods

### General information

Elderly patients regardless of gender, aged 65–81 years, ASA class II or III, who underwent thoracoscopic lung segmental resection in our hospital from January 2023 to December 2023 were retrospectively selected. General anaesthesia and nerve block operations performed by the same anaesthesiologist. They were divided into 2 groups according to the nerve block modality: ultrasound-guided thoracic paravertebral nerve block(Group T) and anterior serratus plane block combined with intercostal nerve block (Group S).

#### Exclusion criteria

History of allergy to local anaesthetic drugs; patients with history of severe liver and kidney diseases; patients with history of heart disease such as severe heart valve disease, cardiac insufficiency, malignant arrhythmia and myocardial infarction; patients with history of alcoholism, drug addiction, epilepsy or mental illness; and patients transferred to open chest during thoracoscopic surgery.

### Anaesthesia methods

**Fig. 1:**
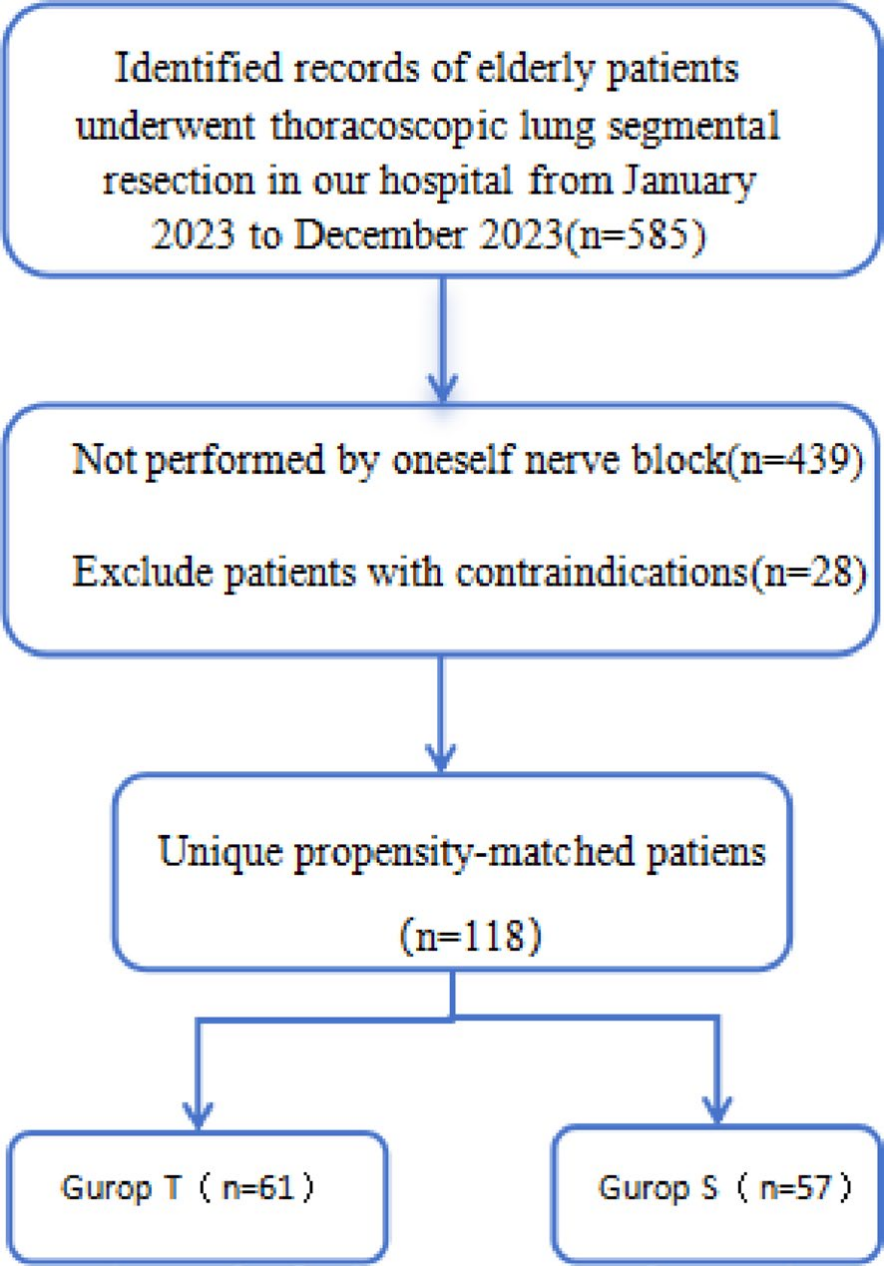
Flow diagram of case selection

After the patients were admitted to the operating theatre, routine cardiac monitoring was performed, peripheral venous access was opened, and sodium acetate Ringer’s solution was titrated (restrictive infusion strategy). Radial artery puncture and cannulation under local anaesthesia and ultrasound-guided right internal jugular vein puncture and cannulation were performed respectively, and invasive arterial pressure and central venous pressure (CVP) were monitored respectively. In both groups, anaesthesia was induced after oxygen administration by face mask (FiO_2_ 100%), midazolam 0.05 mg/kg, propofol 1 mg/kg, sufentanil 0.4ug/kg, and rocuronium bromide 0.6 mg/kg, were used to facilitate endotracheal intubation. All patients were intubated with a left-sided double-lumen endotracheal tube, and the position was confirmed using fiberoptic bronchoscopy to ensure effective bilateral lung isolation. Mechanical ventilation volume control mode (VT 5–6 ml/kg, f 14–16 beats/min, I:E = 1:2, PEEP 5 cmH2O, FiO2 100%).Intraoperative continuous intravenous pumping of propofol 3–5 mg/(kg.h) and dexmedetomidine 0.2-0.2.5ug/(kg.h) to maintain Nactrend 40–50, pumping remifentanil 0.10-0.10.15ug/(kg.min) to maintain analgesia, maintain the appropriate depth of anesthesia, depending on the fluctuation of blood pressure to be the appropriate vasoactive drugs (to maintain the fluctuations in blood pressure). (Maintain blood pressure fluctuation ± 20% of the basal blood pressure) During the operation, if the decrease of blood pressure is more than 20% of the basal value, the patient should be injected with 0.25–0.50.25.50 mg of Metaraminol to maintain the stability of the patient’s intraoperative hemodynamic blood flow. Depending on the experience of the anesthesiologist, additional rocuronium bromide was added to maintain appropriate inotropic relaxation.Intraoperative heat preservation measures were given, and protective one-lung ventilation. After the operation, the patients were awake, muscle strength recovered well, and the exhalation of the double-lumen bronchial catheter should be removed, and airway secretions should be cleared in a timely manner. Observe the vital signs are stable, steward score is greater than 6 points out of the operating room into the ICU to continue to monitor the treatment. Postoperative analgesia: Both groups used intravenous self-control analgesia (PCIA) after surgery, the dose of analgesic drugs was 800 mg of tramadol hydrochloride, 10 mg of tropisetron diluted in saline to 100 mL, the background dose was set at 2 mL/h, the single self-control dose was 0.5 mL, and the locking time was set at 15 min.Remedial analgesic regimen: When the pain visual analog scale score (VAS score) was ≥ 4, patients were given oxycodone hydrochloride injection 3 mg intravenously for analgesia in the ICU. (Fig. 1).

### Nerve block method

In both groups, after induction of anaesthesia, the affected side was turned into a lateral position with the operative side on top. Both underwent nerve block under ultrasound guidance.

**Fig. 2:**
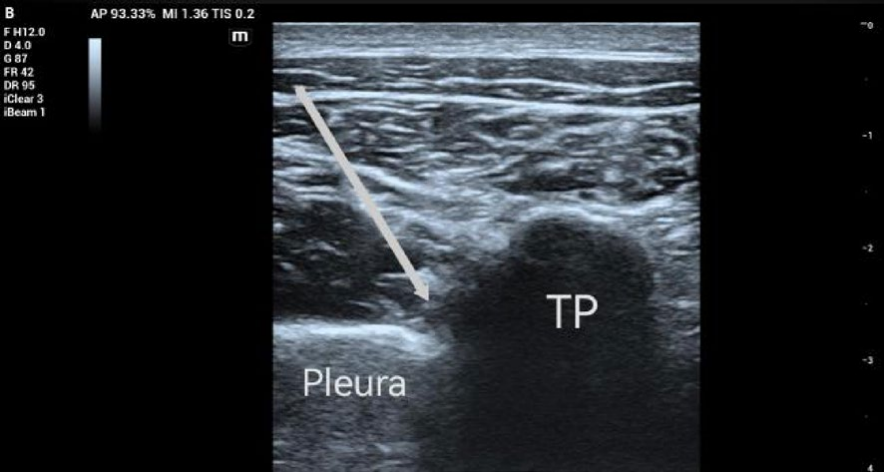
The white arrow indicates the direction and injection site of the needle tip

T group, palpation to determine the position of the T5 spinous process, the ultrasound probe was placed in the vertical posterior midline of the T5 spinous process, slowly sliding the probe to the posterior lateral until the ultrasound image showed an awake thoracic paraspinal interspace, fixed probe in-plane needle, push 2 ml physiological saline to experiment with the appropriate position and then slowly pushed 30 ml of 0.375% ropivacaine, the visible wall layer of the pleura was pressed down. (Fig. 2).

**Fig. 3:**
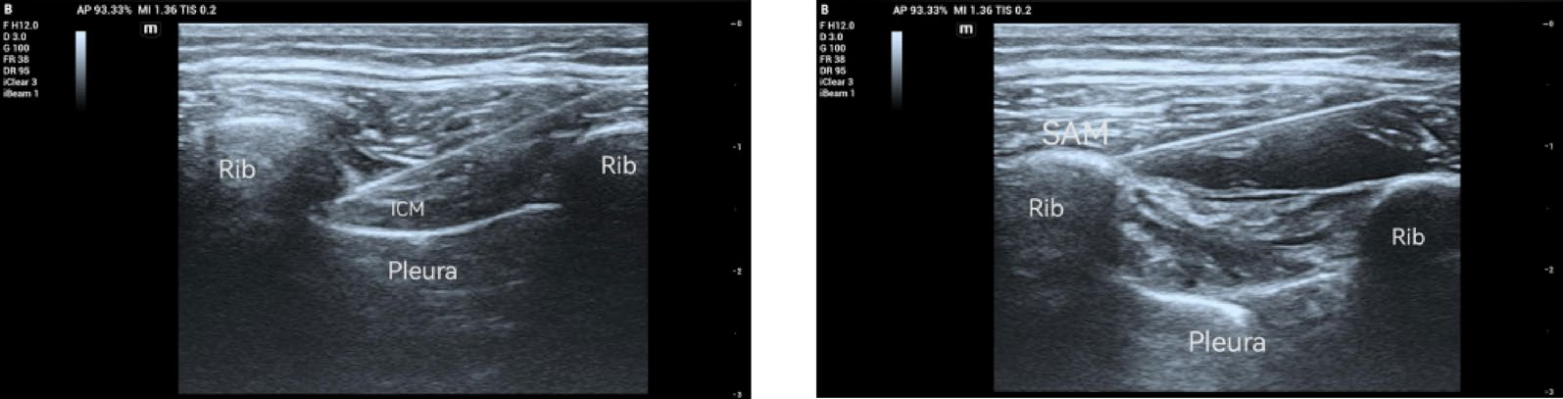
Perform intercostal nerve block first, followed by serratus anterior plane block

Patients in group S took the affected side on the upper, ultrasound probe in the mid-axillary line near the surgical incision adjacent to the rib sweep (usually the 4th or 5th intercostal space), the lower edge of the rib was placed in the centre of the scanning plane, and the ultrasound parameters were adjusted to clearly display the structures of the latissimus dorsi muscle, serratus anterior muscle, ribs and pleura. In the intercostal space where the surgical incision was made, in-plane needle insertion method was adopted, and after the puncture needle reached the fascia between the rib and the serratus anterior muscle, no blood was withdrawn, and a small amount of saline was injected to confirm that the position was satisfactory, and then 7 ml of 0.375% ropivacaine was injected, which was seen to be separated by the drug between the serratus anterior muscle fascial layer and the rib bone; then the needle was withdrawn into the subcutaneous tissues, and the angle of the needle insertion was adjusted and the needle was inserted again into the lower edge of the rib (rib bone where the surgical incision was made). After the incision was made, an intercostal nerve block was performed (3 ml of ropivacaine of the same concentration was injected), and it could be seen that the internal intercostal muscles were separated from the innermost intercostal muscles by the drug.Anterior serratus and intercostal nerve blocks were then performed at one rib segment above and below the surgical incision, for a total of three intercostal segments, with a total dosage of 30 ml of 0.375% ropivacaine. (Fig. 3).

### Outcome measurements

The primary outcome were VAS scores (0–10, 0 = no pain, 10 = worst pain imaginable) at rest and coughing at preoperative (T0), 24 h postoperative (T1), 48 h postoperative (T2) and 72 h postoperative (T3) in both groups.The VAS score was used for both resting pain assessment (resting assessment) and pain assessment when the patient was instructed to cough and inhale deeply (exercise assessment); number of postoperative self-controlled compressions, number of rescue analgesia during the 48 h postoperatively.

The secondary outcomes included basic preoperative data; intraoperative operation time, anesthesia emergence time, extubation time, intraoperative rehydration volume, bleeding volume, urine volume, intraoperative remifentanil consumption dosages and numbers of vasoactive drugs; the occurrence of nausea, vomiting and respiratory depression vomiting and respiratory depression within 48 h, and the occurrence of the complications caused by the puncture (hematoma, pneumothorax, and poisoning of local anesthesia).

Anesthesia emergence time was defined as the interval from discontinuation of anesthetic agents to recovery of spontaneous respiration, eye opening, or following verbal commands.

### Sample size calculation

Based on the sample size calculation formula:: $\:{\text{n = }}\frac{{{\mathrm{2}} \times {\mathrm{(Z}}_{{{\text{1 - a/2}}}} {\text{ + Z}}_{{{\text{1 - }}\beta }} {\mathrm{)}}^{{\mathrm{2}}} \times \sigma ^{{\mathrm{2}}} }}{{\delta ^{{\mathrm{2}}} }}$, where (α = 0.05, two-tailed test), corresponding to $\:{\mathrm{Z}}_{\mathrm{1-a/2}}$=$\:{\mathrm{Z}}_{\mathrm{0.975}}$≈1.96;β = 0.1 (statistical power 90%), corresponding to $\:{\mathrm{Z}}_{{{\text{1 - }}\beta }}$=$\:{\mathrm{Z}}_{\mathrm{0.8}}$≈1.28༛According to references [2, 7] δ = 1(anticipated effect size) andσ = 1.3 (standard deviation) were substituted into the calculation: $\:{\text{n = }}\frac{{{\mathrm{2}} \times {\mathrm{(1}}{\text{.96 + 1}}{\mathrm{.28)}}^{{\mathrm{2}}} \times {\mathrm{1}}{\mathrm{.3}}^{{\mathrm{2}}} }}{{{\mathrm{1}}^{{\mathrm{2}}} }}{\text{ = 35}}{\mathrm{.482}}$ (rounded up).The minimum sample size per group was determined to be 36 subjects. Considering an anticipated attrition rate (As this is a retrospective study, cases with incomplete records or other difficulties in retrieval were anticipated, hence an attrition rate of 30% was estimated, meaning a retention rate of 70%), the initial sample size was adjusted to ensure an adequate number of valid data points: Initial sample size = Calculated sample size/Retention rate ≈ 52.

Therefore, 52 subjects per group needed to be retrospectively reviewed (52 in Group T, 52 in Group S), requiring a total sample size of 104 subjects to compensate for potential attrition. The actual number of cases identified was slightly higher than the calculated initial sample size. Consequently, all actual cases that met the criteria were included in the study. Ultimately, the final sample size was (61 cases in Group T, 57 cases in Group S).

### Statistical processing

SPSS 23.0 statistical software was used for data processing. If the data were continuous, they were expressed as mean ± standard deviation, and firstly, the normality test was performed, and if the normality was satisfied, the two independent samples t-test was used for comparison. For categorical data, the chi-square test was used for unordered outcomes, and the Wilcoxon rank sum test was used for ordered data. Comparison of resting and exercise VAS scores at each time point was first performed by Mauchly’s spherical hypothesis test, and if the spherical hypothesis was satisfied, one-way repeated measures ANOVA was used; if the spherical hypothesis was not satisfied, one-way repeated measures ANOVA was performed after correction by the Greenhouse-Geisser method. *p* < 0.05 was considered to be statistically significant.

## Results

### Primary endpoints

**Fig. 4:**
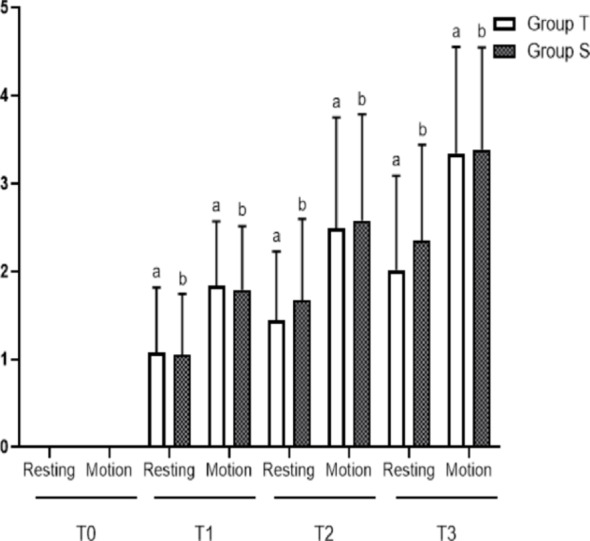
Comparison of resting and motion VAS scores at each postoperative time point between the two groups. a Compared with T0 resting VAS scores, the difference was statistically significant (P<0.05); b Compared with T0 exercise VAS scores, the difference was statistically significant (P<0.05)

The resting and motion VAS scores of the two groups were compared at each time point, and the resting and motion VAS scores of the two groups at T1, T2 and T3 were higher than those at T0, and the differences were statistically significant (*P* < 0.05). Further comparison of the resting and motion VAS scores of the two groups at each time point showed that the difference between the resting VAS scores of group T and group S at each time point was not statistically significant, and the difference between the motion VAS scores of the two groups at each time point was also not statistically significant (*P* > 0.05). (Fig. 4).

There was no statistically significant difference in the comparison of the number of postoperative self-controlled compressions, but there was a difference in the number of postoperative remedial analgesia in group T compared with group S (*P* < 0.05) (Table 1).

**Table 1:** Comparison of the number of postoperative self-controlled compressions and the number of postoperative remedial analgesia

Group	Number of self-controlled compressions/[times (%)]	Number of remedial analgesia/[times (%)]
T	9(14.75)	15(24.59)
S	7(12.28)	6*(10.52)
*t*/^2^	0.154	3.984
*P*	0.695	0.046

Compared with Group T, *P < 0.05

### Secondary endpoints

There was no statistically significant difference in the comparison of gender ratio, age, ASA grading ratio, preoperative combined hypertension and diabetes mellitus between the two groups (*P* > 0.05) (Table 2).

**Table 2:** Comparison of preoperative general conditions of patients in the two groups

Group	No. of cases	Male/female (cases)	Age (years)	Weight *(kg)*	ASA Classification (Class II/III)	Hypertension [cases (%)]	Diabetes mellitus [cases (%)]
T	61	28/33	71.77 ± 3.36	62.95 ± 5.86	21/40	26(42.62)	11(18.03)
S	57	22/35	72.02 ± 3.37	60.82 ± 5.46	25/32	22(39.60)	12(21.05)
*t*/^2^		0.644	0.398	2.036	1.102	0.198	0.171
*P*		0.422	0.691	0.044	0.294	0.656	0.679

There was no statistically significant difference in the comparison of operation time, anaesthesia awakening time, extubation time, intraoperative rehydration volume, bleeding volume, urine volume, use of remifentanil between the two groups of patients (*P* > 0.05); the number of times intraoperative vasoactive drugs were used in the patients of group T was more than that of group S, and the difference was statistically significant (*P* < 0.05) (Table 3).

**Table 3:** Comparison of intraoperative conditions between the two groups of patients

Group	Surgical time(min)	Anaesthesia awakening time (min)	Extubation time *(min)*	Rehydration volume (ml)	Bleeding volume(ml)	Urine volume *(ml)*	Sufentanildosage (ug)	Remifentanil Dosage(ug)	Vasoactive medication use/visit
T	65.98 ± 10.44	8.98 ± 1.85	14.25 ± 1.97	1171.31 ± 140.08	54.67 ± 16.95	263.61 ± 37.77	32.79 ± 3.35	313.77 ± 21.07	33
S	66.14 ± 10.00	8.93 ± 1.87	14.23 ± 2.02	1168.42 ± 133.51	54.91 ± 16.57	265.44 ± 39.51	32.25 ± 3.38	314.21 ± 20.35	15
*t*/^2^	0.083	1.026	1.176	0.709	0.078	0.257	0.873	0.115	8.221
*P*	0.934	0.307	0.242	0.480	0.938	0.797	0.385	0.908	0.004

Respiratory depression, pneumothorax and local anaesthetic poisoning did not occur in either group. There was no statistically significant difference in the nausea and vomiting and the occurrence of puncture complications (*P* > 0.05) (Table 4).

**Table 4:** Comparison of postoperative conditions between the two groups of patients

Group	Nausea and vomiting/[cases (%)]	Respiratory depression/[cases (%)]	Number of cases of puncture haematoma/[cases (%)]	Number of cases of pneumothorax/[cases (%)]	Poisoning by local anaesthetics/[cases (%)
T	8(13.11)	0	3(4.92)	0	0
S	7(12.28)	0	1(1.75)	0	0
*t*/^2^	0.018		0.227		
*P*	0.892		0.634		

## Discussion

Although thoracoscopic segmental lung resection has the advantages of smaller surgical incision and higher patient satisfaction than traditional open thoracotomy, it often requires the operator to manipulate the surgical instruments to pull the pulmonary hilum, pulmonary arteries and veins, trachea, bronchus and pericardium, which results in greater surgical irritation. Minimally invasive thoracoscopic surgery is not equal to minimal pain. The incision is usually made between the mid-axillary line and the anterior axillary line of the fourth or fifth intercostal space, which is surrounded by abundant nerves. Therefore, the use of incision protectors, the pressure of thoracoscopic manipulators on the ribs, and the placing of thoracic drainage tubes at the end of the surgery will stimulate the pleura and squeeze the intercostal nerves, which will lead to increased pain in the patients [8, 9].

Along with the rapid development of accelerated rehabilitation surgical concepts, perioperative pain management has become an indispensable and important part. In recent years, multimodal analgesia has become more and more widespread in clinical application, and nerve block is an important technique in multimodal analgesia programme. It can effectively reduce the total amount of intraoperative opioid use, as well as effectively relieve acute postoperative pain and accelerate patient recovery. With the application of ultrasound technology in the perioperative period, visualisation has improved the safety and precision of nerve block, and various nerve block analgesia modalities are increasingly used for analgesia after thoracoscopic surgery. The analgesic effect of thoracic paravertebral nerve block is comparable to that of epidural block, so it is widely used in thoracic surgery and has become the gold standard [10, 11]. The anterior serratus plane block is a new regional block technique that has been favoured by anaesthesiologists at home and abroad in recent years. It has been shown that the range of the anterior serratus muscle block is generally the cortical branch layer of the intercostal nerve, and only the lateral and anterior walls of the chest wall can be blocked. Although the incision of thoracoscopic surgery is small, it will involve the ventral branch of the intercostal nerve, which innervates the internal intercostal muscles and the innermost intercostal muscles [12, 13], and these two muscle groups will be affected by coughing during postoperative rehabilitation in thoracic surgery, and the anterior serratus plane block cannot be involved in this area, this may be one of the reasons why the anterior serratus block is not a substitute for the paravertebral block as reported in the existing literature [14]. So therefore, the anterior serratus plane block combined with an intercostal nerve block row was selected in this retrospective analysis to comprehensively cover the accumulated muscle groups and nerves of the anterior-lateral incision of thoracoscopic surgery, muscle groups and nerves.

The main results analysis showed that there was no significant difference in pain scores between the serratus anterior plane block combined with intercostal nerve block and paravertebral nerve block in elderly patients undergoing thoracoscopic pulmonary segmentectomy during resting and movement states within 48 h after surgery, suggesting that both multimodal analgesia regimens can provide adequate postoperative pain management for elderly patients.This may be due to the use of visualization ultrasound technology before surgery, which enables more precise operation, better block effect, effective preventive analgesia, increased pain threshold in elderly patients, weakened central or peripheral sensitization of the body, thus achieving good analgesic effect on the chest wall.

However, remedial analgesia within 48 h after surgery, opioid use on the paravertebral nerve group increased significantly, indicating that the serratus anterior plane block combined with intercostal nerve block provided better analgesic effects than the paravertebral block within 48 h after surgery.Previous studies have shown that both simple serratus anterior plane block (SAPB) and thoracic paravertebral block (TPVB) can provide effective postoperative analgesia for patients undergoing thoracoscopic surgery, and SAPB offers better analgesic effects than TPVB at 48 h postoperatively [15]. In this study, the combination of serratus anterior plane block and intercostal nerve block provided more comprehensive analgesic coverage and superior postoperative pain relief.Which may be due to the high density of blood vessels in the paravertebral space, in the paravertebral space injection of local anaesthetics is quickly absorbed by the blood vessels, and the effect of the time of action is relatively short-lasting related [16]. In addition, the epidural space and the thoracic paravertebral space are connected with each other through the intervertebral foramen, and the local anaesthetic is widely diffused so that the local anaesthetic drug is metabolised very quickly, whereas the anterior serratus plane block is not easy to diffuse and absorb the local anaesthetic drug, and it can provide a more adequate thoracic analgesia [14]. The study showed that patients in the thoracic paravertebral nerve block group required higher doses of vasoactive drugs intraoperatively compared to the anterior serratus plane block group. This may be related to the surgical position and anatomical location of the thoracic paravertebral block. Local anesthetics might diffuse into the epidural space, directly affecting spinal nerves and sympathetic nerves. The resulting hemodynamic fluctuations were more significant, with a higher incidence of hypotension, requiring more fluid infusion or administration of vasoactive drugs for symptomatic management [17]. However, due to the special characteristics of lung surgery, intraoperative anaesthesia management will adopt a restrictive fluid infusion strategy to avoid the occurrence of pulmonary oedema and pulmonary complications caused by excessive fluid input, which will affect the patient’s prognosis and recovery [18, 19]. Comparatively speaking, the number of vasoactive drugs applied in the anterior serratus plane block combined with intercostal nerve block group was relatively low, and the intraoperative hemodynamics of the patients were smoother.

This retrospective study also has shortcomings. The VAS score for evaluating the degree of postoperative pain of patients is subjective, with large individual differences, and does not objectively and accurately reflect the degree of pain of patients. Therefore, some objective evaluation factors should be added, such as the inflammatory factors produced by pain stimulation, such as IL-6 and IL-10. It is expected that later multi-centre and large-sample studies will address the optimal injection method and optimal dosage of local anaesthetic for superficial plane nerve block of the serratus anterior muscle. In the future, a systematic review and meta-analysis of postoperative analgesic techniques for thoracic surgery will be conducted.

In conclusion, both ultrasound-guided thoracic paravertebral nerve block and anterior serratus plane block combined with intercostal nerve block can provide effective postoperative analgesia for elderly patients undergoing thoracoscopic pulmonary segmentectomy, serving as safe and feasible multimodal analgesia protocols.Patients in the serratus anterior plane block combined with intercostal nerve block group maintained relatively stable intraoperative hemodynamics and reduced opioid consumption at 48 h postoperatively.Compared to paravertebral nerve block, the anterior serratus plane block combined with intercostal nerve block is advantageous due to its relative ease of application.

## Data Availability

No datasets were generated or analysed during the current study.
